# Behavioral, Physiologic, and Habitat Influences on the Dynamics of *Puumala virus* Infection in Bank Voles (*Clethrionomys glareolus*)

**DOI:** 10.3201/eid0809.010537

**Published:** 2002-09

**Authors:** Sophie Escutenaire, Patrice Chalon, Florence de Jaegere, Lucie Karelle-Bui, Georges Mees, Bernard Brochier, Francine Rozenfeld, Paul-Pierre Pastoret

**Affiliations:** * Pasteur Institute, Brussels, Belgium; † University of Liège, Liège, Belgium; and ‡University of Brussels, Brussels, Belgium

**Keywords:** Hantavirus infections, *Microtinae*, epidemiology, longitudinal studies, behavior, animal, ecology, Belgium

## Abstract

Populations of bank voles (*Clethrionomys glareolus*) were monitored during a 4-year study in southern Belgium to assess the influence of agonistic behavior, reproductive status, mobility, and distribution of the rodents on the dynamics of *Puumala virus* (abbreviation: PUUV; genus: *Hantavirus*) infection. Concordance was high between data from serologic testing and results of viral RNA detection. Wounds resulting from biting or scratching were observed mainly in adult rodents. Hantavirus infection in adults was associated with wounds in the fall, i.e., at the end of the breeding season, but not in spring. In addition, sexually active animals were significantly more often wounded and positive for infection. Hantavirus infection was associated with higher mobility in juvenile and subadult males. Seroconversions observed 6 months apart also occurred more frequently in animals that had moved longer distances from their original capture point. During nonepidemic years, the distribution of infection was patchy, and positive foci were mainly located in dense ground vegetation.

Hantaviruses (family *Bunyaviridae*) are rodent-borne zoonotic agents responsible for human diseases called hemorrhagic fever with renal syndrome (HFRS) in Europe and Asia and hantavirus pulmonary syndrome (HPS) in the Americas (1,2). Viral transmission occurs through inhalation of aerosols from the urine, saliva, or feces of infected rodents and possibly through biting (3–5). Hantavirus infection persists in reservoir species apparently without causing clinical signs (6). In experimentally infected rodents, the virus is distributed in different organs (including lungs, kidneys, intestines, and salivary glands) and elicits the production of antibodies that may be detected lifelong, although the viremia is generally transient (4,7,8). In the wild, adult rodents are generally more often infected than younger animals. The age-dependent prevalence may result from protection of newborns by maternal antibodies and from higher risk of infection for sexually mature rodents through fighting, mating, or communal nesting (9–12). In Europe, *Puumala virus* (PUUV), which causes a mild form of HFRS in humans, is carried by bank voles (*Clethrionomys glareolus*) (13). No data are available on the dynamics of PUUV infection in bank vole populations according to behavioral patterns. Most seroconversions recorded in a capture-mark-recapture (CMR) study of PUUV transmission occurred during the breeding season and in sexually mature voles, with a prevalence bias in favor of mature males (10). Aggressive encounters in adults and the occupation of exclusive territories by breeding females are characteristic of the breeding season in bank voles (14–16).

We studied the influence of aggressive behavior, reproductive status, and mobility of bank voles on the prevalence of PUUV infection. Along with behavioral and physiologic factors, we studied the influence of habitat on bank vole distribution. Two HFRS outbreaks were reported in Belgium in 1996 (224 cases) and in 1999 (124 cases) (17,18). Our survey was conducted from 1996 to 1999 in southern Belgium, where most patients had been reported during the epidemic years. In our trapping sites, rodent population densities were the highest in 1996 and 1999, as was the prevalence of PUUV infection, with 41 (19.2%) of 213 and 259 (39.3%) of 659, respectively, of bank voles positive (19; S. Escutenaire, unpub. data).

## Materials and Methods

### Study areas

From 1996 through 1999, trapping was conducted twice a year (October–November and April–May) at 21 sites distributed in five localities of southern Belgium. All sites were located in broad-leaved or mixed pine and broad-leaved forests. Four of the 21 trapping sites, at Thuin, Montbliart, Momignies, and Couvin, were selected for a CMR survey (19). Each CMR site contained mapped areas with dense or low ground vegetation. The dense ground vegetation included thickets of brambles (*Rubus* sp.), shrubs (including *Corylus avellana*, *Sambucus racemosa*, *Prunus spinosa*, *Cytisus scoparius*, *Crataegus monogyna*, *Salix caprea,* and *Lonicera periclymenum*) and dense populations of plants such as *Pteridium aquilinum* and *Epilobium angustifolium*. The low ground vegetation comprised herbs (*Carex pilulifera*, *Anthoxanthum odoratum*, *Luzula pilosa*, *Dryopteris carthusiana*, *Teucrium scorodonia*, *Scrophularia nodosa,* and *Silene dioica*) or sparse brambles located under dense spruce (*Picea abies*) or oak (*Quercus robur*, *Q. petraea*) foliage.

### Sampling Procedure

On each CMR site, we constructed a 10 X 10 grid of 100 live traps (Sherman Live Trap Co., Tallahassee, FL; Tomahawk Live Trap Co., Tomahawk, WI) spaced at 10-meter intervals. Traps were set for four consecutive nights. Rodents were anesthetized with isoflurane (Forène, S.A. Abbott, Louvain-La-Neuve, Belgium), individually marked by toe-clipping, and released at their original place of capture after a blood sample was collected from the retroorbital sinus. Organs (lungs, liver, kidneys, and spleen) were also collected from any animals found dead in traps. In spring 1999, 120 traps were added to expand the CMR grids, as described (19). On the 17 trapping sites where CMR was not done, 23 to 30 live traps were placed along transects at 5-m intervals for two to four consecutive nights. The trapped animals were humanely killed, and their blood and organs were collected.

### Data Collection

All rodents were examined for sex and weight. Pregnant and lactating females and males with testicles in scrotal position were considered sexually active. Weight limits to distinguish adult, subadult, and juvenile categories were inferred from the analysis of prevalence of infection and reproductive status, according to body mass of rodents. Mass classes, which differed over time (19), were <16 g (juveniles), 16–18 g (subadults), and >18 g (adults) in fall 1996, in spring 1997 and in 1999, and <13 g (juveniles), 13–15 g (subadults), and >15 g (adults) in fall 1997 and in 1998. From spring 1997 on, we recorded the presence of wounds associated with a bite or a scratch on the head (ear perforation or muzzle injuries) of bank voles.

### Serologic Screening and Viral RNA Detection

Sera of rodents trapped from fall 1996 to fall 1998 were screened by an immunoglobulin (Ig) G enzyme-linked immunosorbent assay (ELISA), with PUUV CG18-20, *Hantaan virus* (HTNV) 76-118, and *Dobrava-Belgrade virus* (DOBV)–infected Vero E6 cell lysates as viral antigens (19). Sera collected in 1999 were screened by using a PUUV IgG ELISA kit (Progen Biotechnik, Heidelberg, Germany) (19).

Viral RNA was detected in ground-up lungs by means of reverse transcription (RT) polymerase chain reaction (PCR) test (19). Two nested PCRs with S- or M-segment oligonucleotides were done to amplify the cDNA. The expected size of the amplified fragments was 205 base pairs (bp) (nucleotide [nt] 1033–1237) and 310 bp (nt 2463–2772) for the S (small) and M (medium) genomic segments, respectively.

### Data Analysis

The total number of rodents marked and released during a trapping session was used as an indicator of population size on each CMR site. Capture points of all rodents trapped in the grids were mapped. According to the distribution, we determined the pairs of adult voles with overlapping home ranges. Home range overlap was considered when rodents were captured at the same trap station or when the areas enclosed by the capture points overlapped (20,21). Distances between trapping points of recaptured voles were measured to estimate rodent movements.

Statistical analysis of data was done by chi-square test, Pearson correlation, and Student's *t* test. Pairs of adult rodents with overlapping home ranges were analyzed according to sex and serologic or PCR status. Data for each criteria were processed as binomial distributions.

## Results

### Serologic and RT-PCR Data

IgG antibodies against PUUV were detected in 318 (26.3%) of 1210 bank vole sera collected from fall 1996 through fall 1999. PUUV genomic fragments were amplified in 38 (13.7%) of 277 lung samples. Of 179 bank voles tested both by ELISA and RT-nested PCR, 169 (94.4%) had concordant results. Viral RNA was detected in the lungs of 19 (73.1%) of 26 seropositive animals and in three seronegative ones. As serum and tissues were not available for all rodents and as the concordance between results of serologic testing and PCR was high, data from both tests were pooled for further analysis. Rodents positive by ELISA or PCR or both were considered positive for PUUV infection.

### Frequency of Wounds

Trapping data from spring 1997 through fall 1999 showed that the proportion of wounded bank voles was significantly (chi square 4.73, p=0.03) higher in autumn (150 [25.3%] of 593) than in spring (99 [19.8%] of 501). Adults were significantly (p<0.01) more often injured than juveniles and subadults in 1998 and 1999 (Table 1). The proportion of injured subadults (42 [18.5%] of 227) was also significantly (chi square 12.63; p<0.01) higher than that of wounded juveniles (20 [7.7%] of 259) in 1998 and 1999. Although no difference was observed between either sex in 1997 and 1998, the proportion of wounded animals during the 1999 epidemic year was significantly (p=0.03) higher in females than in males (Table 1).

**Table 1:** Relationship between frequency of wounds and the age and sex of bank voles

Trapping season	J+S^a^ % wounded (total no. of animals)	A % wounded (total no. of animals)	p value	M % wounded (no. of males)	F % wounded (no. of females)	p value
Spring 1997	7.7 (13)^b^	3.4 (29)		8.3 (24)	0.0 (18)	
Fall 1997	3.1 (65)	9.7 (103)		7.8 (116)	5.8 (52)	
Total 1997	3.8 (78)	8.3 (132)		7.9 (140)	4.3 (70)	
Spring 1998	0.0 (2)	35.6 (45)		33.3 (27)	35.0 (20)	
Fall 1998	14.3 (56)	58.5 (123)	p<0.01	46.7 (75)	43.3 (104)	
Total 1998	13.8 (58)	52.4 (168)	p<0.01	43.1 (102)	41.9 (124)	
Spring 1999	6.7 (223)	34.9 (189)	p<0.01	16.2 (222)	23.7 (190)	
Fall 1999	19.0 (205)	46.3 (41)	p<0.01; χ^2^=14.15	20.8 (125)	26.4 (121)	
Total 1999	12.6 (428)	37.0 (230)	P<0.01	17.9 (347)	24.8 (311)	p=0.03; χ^2^=4.67

^a^ J+S, juveniles and subadults; A, adults; M, males; F, females.

### Association between Wounds and Prevalence of Infection

When the fall prevalence rates were compared within mass classes, injured adults were significantly (p<0.01) more often positive than nonwounded ones, although no difference in prevalence was observed in the spring (Table 2). The prevalence in 1999 was significantly (chi square 12.14; p<0.01) higher in subadults (61 [31.4%] of 194) than in juveniles ≥13 g (25 [15.5%] of 161). Subadults were also more often wounded.

**Table 2:** Prevalence of *Puumala virus* infection in adult bank voles, by presence of wounds

Trapping season	Wounded % positive (no. tested)	Not wounded % positive (no. tested)	p value
Spring 1997	0.0 (1)	21.4 (28)	
Fall 1997	20.0 (10)	8.6 (93)	
Spring 1998	6.3 (16)	6.9 (29)	
Fall 1998	15.3 (72)	5.9 (51)	
Spring 1999	68.2 (66)	68.3 (123)	
Fall 1999	68.4 (19)	36.4 (22)	p=0.04; χ^2^=4.19
Spring (1997–1999)	55.4 (83)	51.1 (180)	
Fall (1997–1999)	25.7 (101)	11.4 (166)	p<0.01; χ^2^=9.16

Despite the difference in frequency of wounds between males and females in 1999, the prevalence of infection in adult females (79 [62.7%] of 126) was not higher than in adult males (71 [67.6%] of 105). During the fall of the nonepidemic years (1997 and 1998), the proportion of positive adult males (18 [14.5%] of 124) was significantly higher (chi square 4.40; p=0.04) than that of adult females (6 [5.9%] of 102) although no significant difference in wound frequency was observed between either sex.

### Influence of the Reproductive Status of Adults

**Figure 1:**
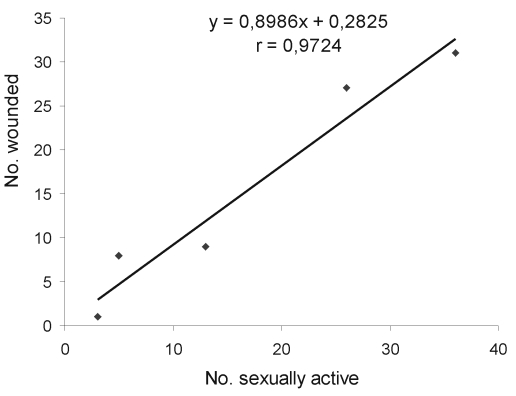
Linear regression in bank voles between the number wounded and the number of sexually active adult males. Significance of correlation: p<0.01. Data used for the analysis were collected from spring 1997 through spring 1999. The scrotal position of testicles was not inspected in fall 1999.

Data from spring 1997 through spring 1999 showed a correlation (r=0.97; p<0.01) between the number wounded and the number of sexually active adult males (Figure 1). The prevalence of infection was significantly higher (chi square 4.31; p=0.04) in sexually active males (10 [24.4%] of 41) than in other adult males (8 [10.1%] of 79) in fall but not in spring. The analysis of wounded animals also showed that in fall, sexually active males still tended to be more often positive (8 [29.6%] of 27) than the other males (1 [7.7%] of 13).

Pregnant and lactating voles were significantly (chi square 5.47; p=0.02) more often wounded (81 [43.1%] of 188) than other adult females (18 [26.9%] of 67). The prevalence of infection was also significantly (chi square 4.77; p=0.03) higher in breeding females (72 [38.7%] of 186) than in nonbreeding ones (16 [23.9%] of 67). Wounded animals showed no statistically significant difference in prevalence between sexually active (28 [34.6%] of 81) versus sexually inactive adult females (8 [44.4%] of 18).

### Recapture of Rodents

Fifty-three bank voles were recaptured on the grids during subsequent trapping periods. For all but one animal, the recapture occurred within 6 months after the first trapping. Twelve (22.6%) rodents were found positive during both capture sessions, and two (3.8%) 8-g juveniles were seropositive when first trapped and seronegative 6 months later. Twenty-two (41.5%) rodents were negative both times, and 17 (32.1%) seroconverted between the first and the second trapping. Of the 17 rodents that seroconverted, nine were juveniles or subadults at first capture and eight were adults. The proportion of animals that acquired wounds between the spring and the fall tended to be higher in rodents that seroconverted (4 [44.4%] of 9) than in the ones that remained negative (3 [25.0%] of 12), although the difference was not statistically significant (p=0.35).

### Movement within CMR Sites

**Figure 2:**
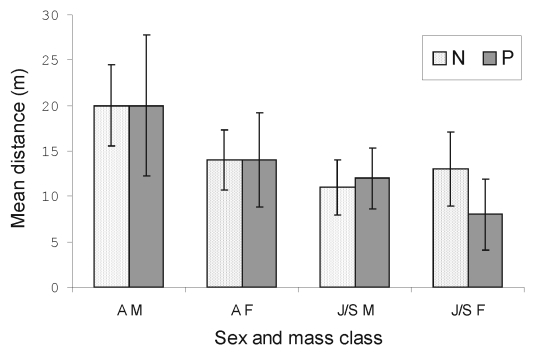
Mean distances (m) between capture points of positive and negative bank voles for PUUV infection in the four capture-mark-recapture (CMR) grids. N, negative; P, positive; AM, adult males; AF, adult females; J/S M, juvenile and subadult males; J/S F, juvenile and subadult females. Error bars represent 95% confidence intervals.

Of the rodents caught more than once during the 4-day sessions, 83 (27.5%) of 302 were trapped at one position only. Adult males were the most mobile, with a mean distance between capture points of 20 m (Figure 2). The distances covered by the positive and the negative bank voles did not differ significantly (Figure 2). However, juvenile or subadult (≥13 g) males that had moved distances of ≥20 m were significantly (chi square 3.86; p=0.05) more often positive than the less mobile ones with prevalence rates of 43.8% (7/16) and 19.6% (11/56), respectively. Mean distance covered was negatively correlated with the population density in adults (correlation coefficient [r] 0.84; p<0.01) and in juveniles and subadults (r 0.93; p<0.01). The rodents were the least mobile during the 1999 epidemic year.

Males that seroconverted between two trapping periods had moved a significantly (Student t value 3.16; p=0.01) longer distance from their original capture point (mean distance 70 m) than males that remained negative (mean distance 29 m). Furthermore, the proportion of males and females that were captured at distances ≥60 m from the original trapping point was significantly (chi square 6.95; p<0.01) higher in the group that seroconverted (9 [52.9%] of 17) than in the group that remained negative (3 [13.6%] of 22).

### Distribution in the CMR Sites

**Figure 3:**
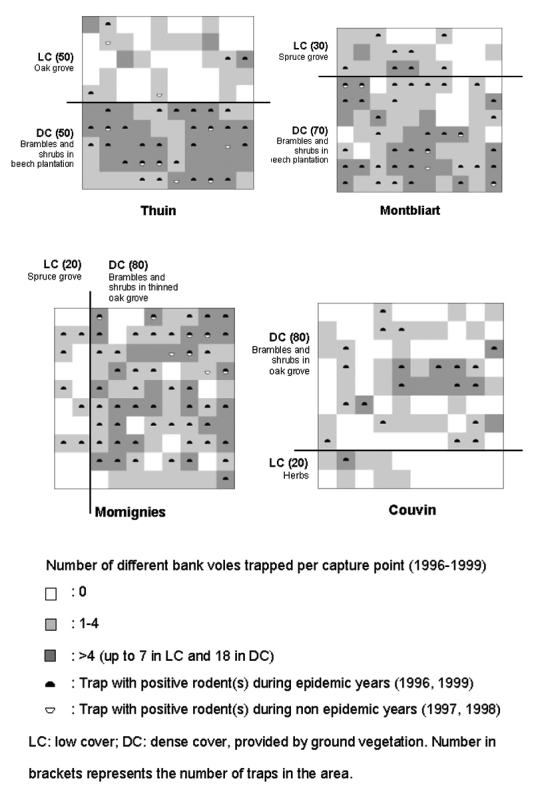
Distribution of trapped bank voles through 1996–1999 and representation of the vegetation cover. On each grid, the 100 live traps are represented by white, light, or dark gray squares. LC, low cover; DC, dense cover provided by ground vegetation. Number in parentheses is the number of traps in the area.

The proportion of captures in the area with dense ground vegetation (Figure 3) was 83.0% (318/383) at Thuin, 87.8% (316/360) at Montbliart, 95.5% (340/356) at Momignies, and 90.8% (118/130) at Couvin. At all sites, the proportion of captures was significantly (p<0.01) higher in dense ground vegetation than in the other part of the grid. Comparison of prevalence of infection between both types of vegetation did not show significantly higher rates in dense ground cover (Table 3). During the nonepidemic years (1997 and 1998), however, positive animals were detected only twice in low ground cover of Thuin but were trapped seven times in dense ground vegetation of Thuin, Montbliart, and Momignies (Table 3, Figure 3).

**Table 3:** Prevalence of *Puumala virus* infection by location and type of ground vegetation

Trapping site	1996	1997		1998		1999	
	F ^a^ % positive (no. tested)	S % positive (no. tested)	F % positive (no. tested)	S % positive (no. tested)	F % positive (no. tested)	S % positive (no. tested)	F % positive (no. tested)
Dense cover							
Thuin	21.2 (66)	20.0 (10)	4.3 (23)	10.0 (10)	0.0 (29)	12.8 (47)	21.5 (65)
Montbliart	7.7 (26)	40.0 (5)	16.7 (30)	50.0 (4)	0.0 (48)	44.9 (49)	27.2 (81)
Momignies	17.9 (28)	0.0 (0)	0.0 (31)	0.0 (14)	15.7 (51)	64.8 (54)	28.3 (60)
Couvin	^—b^	0.0 (1)	0.0 (19)	0.0 (9)	0.0 (17)	59.3 (27)	50.0 (18)
Low cover							
Thuin	40.0 (5)	33.3 (3)	10.0 (10)	0.0 (0)	0.0 (6)	0.0 (13)	17.6 (17)
Montbliart	42.9 (7)	0.0 (2)	0.0 (4)	0.0 (1)	0.0 (3)	50.0 (6)	0.0 (3)
Momignies	50.0 (6)	0.0 (0)	0.0 (1)	0.0 (0)	0.0 (0)	50.0 (4)	33.3 (3)
Couvin	—	0.0 (0)	0.0 (7)	0.0 (0)	0.0 (1)	66.7 (3)	0.0 (0)

^a^F, fall; S, spring. ^b^Trapping in fall 1996 at Couvin was performed on a limited 0.36-ha area, where the vegetation cover was not determined.

Among adult rodents that had overlapping home ranges in the CMR sites were significantly more male pairs (65 of 173) and fewer female pairs (26 of 173) than expected by chance (binomial probabilities<0.01; proportion of males 52.1%). Statistical evidence of association was found between the home range overlap and the serologic and PCR status for PUUV infection. The number of pairs including two positive animals (24/173) was higher and the number of pairs composed of a positive and a negative animal (41/173) was lower than expected by chance (binomial probabilities<0.01; prevalence of infection 22.2%).

## Discussion

A widespread distribution of hantavirus infection has commonly been reported in rodent hosts during HFRS and HPS epidemics (19,22,23). Transmission of the virus is higher after sudden increases in rodent population density, assumed to be related to favorable ecologic conditions (profusion of food or mild weather) (24,25). In some field studies, the prevalence of infection in rodents during non epidemic periods did not appear to be immediately related to population density (19,26–29). Social behaviors and habitat features have been proposed as additional factors influencing the dynamics of infection in the wild (29–32). In rats (*Rattus norvegicus*) and deer mice (*Peromyscus maniculatus*), which are the hosts of *Seoul virus* (SEOV) and *Sin Nombre virus* (SNV), respectively, agonistic encounters could play a role in viral transmission, as suggested by the higher prevalence of infection in wounded adults (9,32–35).

In our study, wounds resulting from biting or scratching were mainly observed in sexually mature bank voles. Wounded adults were more likely than nonwounded ones to be positive for infection in fall (i.e., at the end of the breeding season) but not in spring. Aggressive encounters would therefore play an important role in PUUV transmission during the breeding season, while behaviors such as communal nesting or mutual grooming would be determinant factors in the dynamics of infection during the winter. Aggressive behaviors begin at the onset of sexual maturity and are probably testosterone dependent in males (14). The proportion of injured animals and the prevalence of infection were higher in adult breeding males and breeding females than in other adults. These observations underline the close relationship between aggressiveness and breeding activities, which could be associated risk factors for hantavirus infection. In contrast with results from previous studies of wild rats and deer mice (9,32,33), adult males were not wounded more often than adult females. However, the prevalence of infection was significantly higher in adult males than in adult females during the fall of the nonepidemic years (1997 and 1998). Sex-related physiologic, immunologic, or behavioral characteristics might therefore be additional factors involved in the transmission of infection. In experimentally infected rats, males were more likely than females to shed SEOV in saliva and through multiple routes (urine, feces, and saliva) (36). They were also found to shed virus in saliva and feces longer than did females (37). Although no similar studies have been performed in bank voles, the observations of SEOV in rats could indicate that fighting results in more efficient viral transmission in males than in females, who generally direct their aggression toward female intruders (14). In addition, males frequently spread small quantities of urine and feces to mark their territory and to indicate their social status during the breeding season (38). Thus, olfactory exploration of conspecific shelters and home ranges might also expose males to a higher risk of infection than females.

Movements and distribution of bank voles are also factors involved in the dynamics of PUUV infection. The association of hantavirus infection with longer distances traveled in juvenile and subadult males and in animals captured 6 months apart underlines the importance of mobility in viral transmission. The difference of prevalence between either sex in fall 1997 and 1998 may also be linked to the higher mobility of adult males compared with adult females. Our observations corroborate results from a previous study of deer mice in which adult males living in patchy vegetative habitats were more mobile and also more often infected than adult males in dense shrub habitats (32). However, our data suggest that the mobility of bank voles was the lowest during the epidemic years. The high prevalence of infection during these years may have resulted from the high population densities, which allowed frequent encounters between rodents. Home range overlap among positive adult voles was more frequent than expected by chance. This observation could reflect a focal distribution of infected animals or may indicate that viral transmission occurs more readily between animals sharing parts of their home ranges. Direct and repeated contacts with infected conspecifics or contacts with recently shed infectious urine or feces could represent risk factors for closest neighbors within a wild breeding colony. The frequent overlap of home ranges recorded in adult males may therefore be one of the factors increasing the risk of infection in males when compared with females.

In our four CMR grids, the type of vegetation influenced the distribution of the rodents. During nonepidemic years, positive animals were more frequently trapped in dense ground vegetation where brambles were abundant. If trapping success indicates preferred habitat, then areas with dense ground cover could constitute foci for bank voles, allowing PUUV to persist during the low prevalence periods. A discontinuous distribution of the rodents could limit viral transmission, as suggested by the absence or low number of positive animals in the isolated groups from the low ground cover in Thuin (Figure 3). An uneven distribution of positive rodents related to the vegetation cover has been observed in *Peromyscus* species (28,32,33,39,40) with more restricted and well-defined focal ranges during periods of low population densities (31). A correlation was also found between prevalence of SNV infection and habitat characteristics; negative sites were associated with low and homogeneous vegetation cover (29).

We have suggested that a threshold in population density may be a determinant for the persistence of PUUV infection at a site (19). In this study, behavioral and physiologic factors, such as aggressiveness, mobility and reproductive status of bank voles, were shown to influence the prevalence of PUUV infection. The habitat determines the distribution of the rodents and therefore also constitutes a crucial element influencing the hantavirus enzootic cycle.
